# Neuronal sensitivity to TDP-43 overexpression is dependent on timing of induction

**DOI:** 10.1007/s00401-012-0979-3

**Published:** 2012-04-27

**Authors:** Ashley Cannon, Baoli Yang, Joshua Knight, Ian M. Farnham, Yongjie Zhang, Charles A. Wuertzer, Simon D’Alton, Wen-lang Lin, Monica Castanedes-Casey, Linda Rousseau, Brittany Scott, Michael Jurasic, John Howard, Xin Yu, Rachel Bailey, Matthew R. Sarkisian, Dennis W. Dickson, Leonard Petrucelli, Jada Lewis

**Affiliations:** 1 Department of Neuroscience, Mayo Clinic, Jacksonville, FL 32224 USA; 2 Department of Obstetrics and Gynecology, University of Iowa, Iowa City, IA 52242 USA; 3 Department of Neuroscience, University of Florida, Gainesville, FL 32611 USA; 4 Center for Translational Research in Neurodegenerative Disease, University of Florida, Gainesville, FL 32611 USA

**Keywords:** Amyotrophic lateral sclerosis, Apoptosis, Frontotemporal lobar degeneration, Neurodevelopment, TDP-43, Transgenic mice

## Abstract

Ubiquitin-immunoreactive neuronal inclusions composed of TAR DNA binding protein of 43 kDa (TDP-43) are a major pathological feature of frontotemporal lobar degeneration (FTLD-TDP). In vivo studies with TDP-43 knockout mice have suggested that TDP-43 plays a critical, although undefined role in development. In the current report, we generated transgenic mice that conditionally express wild-type human TDP-43 (hTDP-43) in the forebrain and established a paradigm to examine the sensitivity of neurons to TDP-43 overexpression at different developmental stages. Continuous TDP-43 expression during early neuronal development produced a complex phenotype, including aggregation of phospho-TDP-43, increased ubiquitin immunoreactivity, mitochondrial abnormalities, neurodegeneration and early lethality. In contrast, later induction of hTDP-43 in the forebrain of weaned mice prevented early death and mitochondrial abnormalities while yielding salient features of FTLD-TDP, including progressive neurodegeneration and ubiquitinated, phospho-TDP-43 neuronal cytoplasmic inclusions. These results suggest that neurons in the developing forebrain are extremely sensitive to TDP-43 overexpression and that timing of TDP-43 overexpression in transgenic mice must be considered when distinguishing normal roles of TDP-43, particularly as they relate to development, from its pathogenic role in FTLD-TDP and other TDP-43 proteinopathies. Finally, our adult induction of hTDP-43 strategy provides a mouse model that develops critical pathological features that are directly relevant for human TDP-43 proteinopathies.

**Electronic supplementary material:**

The online version of this article (doi:10.1007/s00401-012-0979-3) contains supplementary material, which is available to authorized users.

## Introduction

TAR DNA binding protein (TDP-43), encoded by the *TARDBP* gene on human chromosome 1, is a major pathological component of the neuronal inclusions associated with amyotrophic lateral sclerosis (ALS) and frontotemporal lobar degeneration (FTLD-TDP) [28]. The neuropathology of these conditions is characterized by ubiquitin- and TDP-43-positive neuronal and glial cytoplasmic inclusions, neuronal intranuclear inclusions, and dystrophic neurites. The majority of ALS cases have TDP-43 pathology, except for cases caused by either SOD1 or FUS mutations [28]. *TARDBP* mutations have been identified in ALS, but they only account for 4 % of familial and 1.5 % of sporadic cases [25]. FTLD-TDP is the most common FTLD subtype accounting for nearly 50 % of all cases [8, 12], but only three FTLD patients have been identified with *TARDBP* sequence variants [5, 6, 20]. Recently, expansion repeats in the *C9ORF72* gene have been identified as the most common genetic abnormality in familial FTLD-TDP and ALS [13, 30]. TDP-43 pathology in the absence of *TARDBP* mutations is also found in hippocampal sclerosis of the elderly, as well as a subset of patients with Alzheimer’s disease, Parkinson’s disease or other neurodegenerative disorders [2, 3, 15, 17, 18, 29, 37], suggesting a pervasive involvement of TDP-43 in neurodegeneration. Therefore, pathology with wild-type TDP-43 is associated with a range of both primary and secondary TDP-43 proteinopathies.

TDP-43 plays a role in transcription and splicing regulation, with the number of target genes constantly growing. Other functional roles that are not well characterized include microRNA processing, RNA transport, cell division, and apoptosis [7]. It is currently unclear if TDP-43 promotes neurodegeneration through a loss of one or more of these functions or through a toxic gain of function or both. Loss of TDP-43 in mice is lethal at any age [9, 21, 32, 42], supporting loss of function as a potential neurodegenerative mechanism. Conversely, the phenotypes of several transgenic TDP-43 mouse models have been strikingly consistent, including weight loss, gait abnormalities, abnormal hind limb escape reflex, and early lethality [35, 40, 41, 43]. These findings suggest that even low levels of human TDP-43 (hTDP-43) overexpression are pathogenic, regardless of wild type or mutant origin. A number of studies from our lab and others have now shown that murine TDP-43 is reduced in response to the overexpression of exogenous TDP-43; one study reported on a conditional TDP-43 model similar to that utilized for our current study [19]. None of these studies have examined the impact of TDP-43 overexpression on neurons at different stages of development.

Given evidence that TDP-43 may be critically involved in both development and neurodegeneration, we designed a transgenic mouse model, termed iTDP-43_WT_ that conditionally expresses TDP-43 under the control of the tetracycline conditional system of gene regulation [16]. These transgenic mice allowed us to determine if overexpression of hTDP-43 in neurons at different stages of maturation alters the impact of TDP-43. Here, we show that moderate hTDP-43 overexpression within the developing forebrain results in a complex phenotype, including early lethality, early and extensive neuronal loss with apoptosis, perikaryal clusters of abnormal mitochondria and cytoplasmic inclusions of phosphorylated TDP-43 as well as increased ubiquitin immunoreactivity and gliosis. We also show that specific induction of hTDP-43 later in forebrain maturation prevents early lethality, severe early onset neurodegeneration, and mitochondrial abnormalities, yet produces salient features of FTLD-TDP, including progressive neuronal loss, reactive gliosis, and TDP-43 inclusions that co-localize with ubiquitin. These mice now provide a model in which developmental effects of TDP-43 overexpression can be distinguished from degenerative effects of TDP-43 in the mature nervous system.

## Materials and methods

### Ethics statement

All mice were utilized with approval and in accordance with the Mayo Clinic Institutional Animal Care and Use Committee and the University of Florida Institutional Animal Care and Use Committee.

### Transgenic mice

iTDP-43_WT_ mice were generated similarly to a previously described protocol [31]. Full length, untagged, human TDP-43 cDNA, amplified using a TDP-43-myc plasmid as a template [44], was inserted into the inducible expression vector pUHD 10-3 (Hermann Bujard, ZMBH) containing five tetracycline operator sequences. The construct was confirmed by restriction enzyme digest and direct sequencing. The transgenic fragment was obtained by *Bsr*BI digestion, gel purified followed by β–agarase digestion (NEB), filtration and concentration. The modified TDP-43 transgene was injected into the pronuclei of donor FVB/NCr embryos (Charles River). These responder mice were bred with 129S6 mice (Taconic) with the tetracycline transactivator (tTA) transgene downstream of calcium-calmodulin kinase II alpha (CaMKIIα) promoter elements [27] to produce the iTDP-43_WT_ transgenic mice with forebrain hTDP-43 expression. For TDP-43 transgene suppression studies during development, doxycycline water (1.5 g/l doxycycline and 4 % sucrose) was placed in breeding cages for 2 days in addition to doxycycline diet (Harlan; 200 mg/kg), which remained in the breeding cage until pups were weaned at 21 days. Doxycycline, a tetracycline derivative, binds tTA and prevents transgene expression. At 21 days, weanlings were placed in cages with regular chow to allow hTDP-43 transgene expression to commence.

### Immunohistochemistry

After euthanasia via cervical dislocation, brains were harvested and divided along the midline. The right hemisphere was flash-frozen on dry ice, while the left hemisphere was drop fixed in 10 % neutral buffered formalin for histological analyses. Brains were embedded in paraffin and cut into 5-μm sagittal sections. Tissues were immunostained with monoclonal antibodies to TDP-43, pS403/404-phosphorylated TDP-43, ubiquitin, ionized calcium-binding adaptor molecule 1 (Iba1), glial fibrillary acidic protein (GFAP), and cytochrome C oxidase subunit IV (COX-IV), using the DAKO Autostainer (DAKO Auto Machine Corporation, Carpinteria, CA) with DAKO Envision+ HRP System. For cleaved caspase 3 and neuronal nuclear antigen (NeuN) double labeling, the DAKO Envision G2 Double Stain System was used. The peroxidase labeling of cleaved caspase 3 was visualized with diaminobenzidine (DAB), and the alkaline phosphatase labeling of NeuN was detected with Vector Blue alkaline Phosphatase Substrate Kit III (Vector Laboratories, Burlingame, CA). See Supplementary Table 1 for a complete list of primary antibodies used. Hematoxylin and eosin (H&E) staining was performed by standard procedures.

### Protein isolation and Western blotting

Brains were weighed and homogenized in lysis buffer (50 mM Tris base, 274 mM NaCl, 5 mM KCl pH 8.0, 1 % Triton X-100, 2 % SDS, and protease and phosphatase inhibitors) at 1 ml/100 mg. Samples were sonicated and centrifuged at 16,200*g* for 20 min at 20 °C. Supernatant protein concentration was determined by BCA assay (Pierce). Approximately, 50 μg protein was loaded onto 10 % Tris–glycine polyacrylamide gel (Novex). After electrophoresis, gels were transferred to a nitrocellulose membrane for 2 h at 200 mA constant current. Membranes were blocked with 5 % milk in Tris-buffered saline and 0.1 % Triton X-100 (TBS-T; Sigma-Aldrich) for 1 h and probed overnight at 4 °C with one of the following primary antibodies in 5 % TBS-T: mouse monoclonal TDP-43 antibody recognizing human TDP-43, rabbit polyclonal TDP-43 antibody recognizing total TDP-43, and mouse monoclonal glyceraldehyde-3-phosphate dehydrogenase antibody (GAPDH). See Supplementary Table 1 for a complete list of primary antibodies used. The membrane was washed 5 times for 5 min in TBS-T and then incubated in the appropriate secondary antibody for 1 h. The membrane was again washed 5 times for 5 min in TBS-T. ECL reagent was added for 2 min, and the membrane was exposed to X-ray film. For reprobing, the membrane was stripped with 70 mM SDS in Tris HCl (pH 6.8) with 0.7 % BME for 15 min at 55 °C before processing as described above.

### Quantitative PCR

Total RNA was isolated from dissected hippocampus and cortex using TRIzol reagent (Invitrogen) and Pure Link RNA Mini Kit (Invitrogen), and 2 μg were used to synthesize cDNA using the High Capacity cDNA Reverse Transcription Kit (Applied Biosystems). All samples were run in triplicate on the BioRad CFX384 Real-Time PCR Detection System. A dilution series was used to construct a standard curve for each primer pair from which normalization and subsequent fold change calculations were performed. Primer sequences for qPCR were mouse Tardbp F (AAAGGTGTTTGTTGGACGTTGTACAG), mouse Tardbp R (AAAGCTCTGAATGGTTTGGGAATG), Gapdh F (CATGGCCTTCCGTGTTCCTA) and Gapdh R (CCTGCTTCACCACCTTCTTGAT). Single PCR products were verified by melt curve analysis.

### TUNEL staining

The ApopTag Peroxidase In Situ Apoptosis Detection Kit (Chemicon) was used to detect DNA fragmentation by labeling the free 3′-OH termini. Staining was performed according to the manufacturer recommendations. TUNEL-positive cells were visualized by the chromogen 3′-3′ diaminobenzidine (DAB) in sections that were counterstained with methyl green.

### Immunofluorescence staining

Paraffin sections of brain tissue were deparaffinized and rehydrated in a graded series of alcohols. Tissue was blocked in DAKO Protein Block for 1 h then incubated with primary antibodies overnight at 4 °C. See Supplementary Table 1 for a complete list of primary antibodies used. After washing, tissue was incubated in Alexa Fluor 488 and Alexa Fluor 568 secondary antibodies (1:500, Invitrogen) for 1 h at room temperature. Vectashield with DAPI (Vector Laboratories) was used to stain nuclei. Images were captured using an Axio Imager.Z1 microscope (Zeiss).

### Quantification of neurons, TDP-43 inclusions, and caspase 3 immunoreactivity

Three samples per group were chosen and tissue sections approximately 2.0–2.1 mm from the midline were used to quantify the total number of neurons (labeled by NeuN) and the number of neurons containing phospho-TDP-43 and ubiquitin inclusions in addition to neurons with caspase 3 immunoreactivity. Immunofluorescent staining was performed as described above. Images were captured from different cortical regions (primary motor cortex, secondary motor cortex, and primary sensory cortex) per sample after scanning slides at 40× with ScanScope (Aperio). Images were analyzed with ImageScope software (Aperio) for quantification.

## Results

### Continuous hTDP-43 overexpression yields complex pathological profiles

**Fig. 1:**
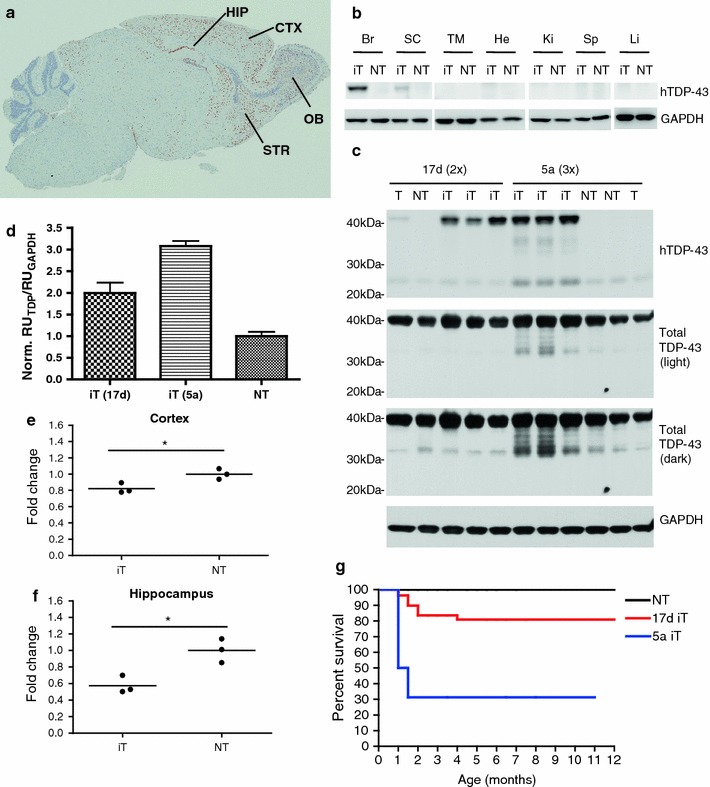
Overexpression of hTDP-43 in the developing forebrain leads to reduced survival and TDP-43 biochemical changes. **a** Immunostaining of a symptomatic 24-day-old 5a bigenic TDP-43/tTA iTDP-43_WT_ mice (iT) sagittal brain section shows an hTDP-43 expression pattern that is anticipated with the CaMKIIα promoter, which includes the cortex (CTX), hippocampus (HIP), olfactory bulb (OB), and striatum (STR). **b** Western blot of tissue lysates from 6-month 17d iT and non-transgenic (NT) mice from brain (Br), spinal cord (SC), thigh muscle (TM), heart (He), kidney (Ki), spleen (Sp), and liver (Li) probed for hTDP-43 reveals expression in the brain with lower levels in the spinal cord of iT mice. **c** Western blot of brain lysates of NT mice, transgenic mice containing only the TDP-43 responder (T), and bigenic TDP-43/tTA mice (iT) from the 17d (2-month old) and 5a (symptomatic, 24-day old) founder lines using antibodies that either detects human TDP-43 (hTDP-43) or both endogenous mouse TDP-43 and hTDP-43 (total TDP-43). GAPDH was used as a loading control. iT mice from founder line 17d have roughly 2× levels of TDP-43 expression when compared to NT controls while iT mice from 5a have roughly 3× overexpression. Minimal leakiness is shown in the TDP-43 only mice from founders 17d and 5a. **d** Densitometric analysis of the relative units of total TDP-43 normalized by the relative units of the GAPDH loading control shown *in panel* (**c**). **e**, **f** Quantitative real time PCR using murine-specific TDP-43 primers demonstrate reduced expression of endogenous TDP-43 in 2-month-old 17d iT cortex (**e**) and hippocampus (**f**) relative to NT littermates, (*n* = 3). **g** A survival curve of the 5a and 17d founder lines shows that iT mice from line 5a (*n* = 16) only has a 30 % survival after 2 months, while the iT mice from line 17d (*n* = 110) has 80 % survival after 2 months compared to NT controls (*n* = 168). SEM shown in **d**. Statistical analysis was assessed by Student’s *t* test in **e**,** f**. *P < 0.05

To generate iTDP-43_WT_ mice, wild-type human TDP-43 (hTDP-43) cDNA was placed behind a minimal CMV promoter with tetracycline operator sequences that effectively blocked human hTDP-43 expression in single transgenic mice (TDP-43_WT_). By crossing these mice with mice expressing the tetracycline transactivator (tTA) under the CaMKIIα promoter [26], we created bigenic iTDP-43_WT_ mice that expressed hTDP-43 in the cortex, hippocampus, olfactory bulb, and striatum, which is the expected expression pattern for this CaMKIIα promoter (Fig. 1a, Supplementary Fig. 1). We further examined hTDP-43 expression in various organs and found only low level expression in the cervical spinal cord (Fig. 1b).

We screened 90 potential founders, identified 7 overexpressing lines and focused our studies on two founder lines, 5a and 17d, that expressed TDP-43 at the highest levels in response to the introduction of the tTA transgene. iTDP-43_WT_ mice derived from founder lines 5a and 17d expressed human TDP-43 protein at 3- and 2-fold, respectively, over endogenous TDP-43 levels in NT brain (Fig. 1c, d). Some variability in hTDP-43 expression was observed in the 17d line (range from 1.6- to 2.4-fold; Fig. 1d). iTDP-43_WT_ mice from both founder lines showed negligible expression of the transgene in the absence of the tTA activator (Fig. 1c). We and others have previously reported that TDP-43 levels appear to be tightly regulated [4, 19, 33, 43], and we found similar results in the iTDP-43_WT_ mice at the RNA level. Compared to NT, mTDP-43 RNA was down-regulated in both the cortex (0.821 ± 0.036; *P* = 0.027) and hippocampus (0.575 ± 0.062; *P* = 0.015) of iTDP-43_WT_ mice (17d) in response to the overexpression of hTDP-43 (Fig. 1e, f). Without a commercially available antibody for mTDP-43, we were unable to specifically assess down-regulation of mTDP-43 at the protein level; however, Igaz and colleagues [19] previously demonstrated that endogenous mouse TDP-43 protein is down-regulated in a similar model system.

Only 30 % of iTDP-43_WT_ mice from the 5a founder line survived past 2 months (2M) of age, while 80 % of iTDP-43_WT_ mice from line 17d survived through the same time point (Fig. 1g). iTDP-43_WT_ mice from both founder lines that failed to survive past 2M were phenotypically similar, showing reduced spontaneous activity, weight, and grooming prior to death (Supplementary Fig. 2). These data demonstrate that survival is dose dependent with respect to hTDP-43 expression.

**Fig. 2:**
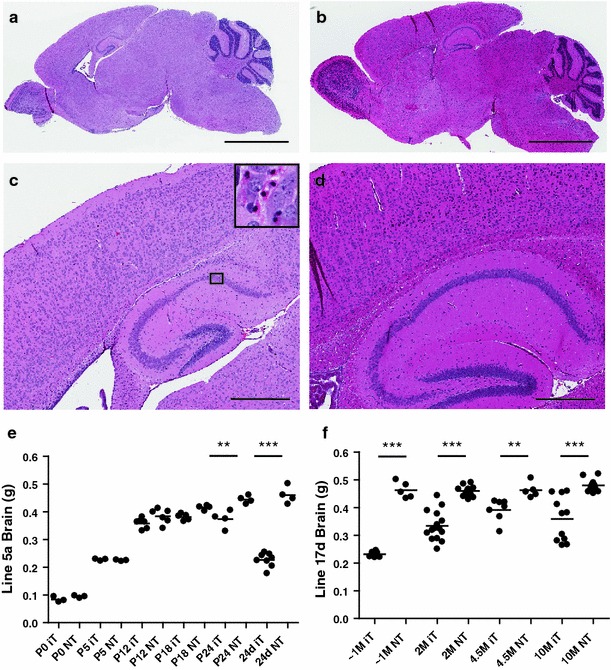
hTDP-43 overexpression in the developing brain results in early and severe neuronal loss. **a**, **b** Hematoxylin and eosin (H&E) staining of sagittal sections from symptomatic 5a iT mice (**a**) shows considerable ventricular enlargement, cortical thinning, and hippocampal atrophy when compared to NT, (**b**) age-matched mice at 24 days of age. **c**, **d** Higher magnification of the sagittal sections highlights the significant ablation of CA1 and dentate gyrus in iT (**c**) compared to NT (**d**) mice. Note the apoptotic bodies (**c**, *inset*) suggesting the onset of apoptotic death in the CA1 of iT mice. **e** Whole brain weights of 5a iT and NT mice at P0, P5, P12, P18 and 24-day old demonstrate that developing iT brains increase in weight normally until P12 but drastically decrease in weight by 24-day old. **f** Whole brain weights of 17d iT mice have significantly lower brain weights than NT mice at all ages examined but are most severe in the symptomatic iT mice approximately 1-M old (range 24–45 days). Of note, the brain weights of 17d iT mice that survived past 2 months of age did not progressively decrease with age. The *bar* represents 2,500 μm in **a**, **b** and 500 μm in **c**, **d**. Statistical analysis was assessed by Student’s *t* test in **e**, **f**. ***P < 0.001, **P < 0.01

Neuronal loss is a prominent feature of TDP-43 proteinopathies such as FTLD-TDP and ALS. We examined the brains of iTDP-43_WT_ mice and found that they had striking forebrain atrophy regardless of phenotype (Fig. 2); however, the extent of atrophy and the relative age at which atrophy occurred were dramatically different than observed in human TDP-43 proteinopathies, with almost complete obliteration of neuroanatomical structures, such as the dentate fascia of the hippocampus. Brains from iTDP-43_WT_ mice exhibit ventricular dilation, cortical thinning, reduced cortical thickness and severe hippocampal atrophy, which contribute to a macroscopic decrease in brain size (Fig. 2a–d). While these features were present in both transgenic lines, brains from symptomatic mice from both lines were roughly twofold smaller than age-matched non-transgenic (NT) mice (Fig. 2e, f). Postnatal brain weights of iTDP-43_WT_ mice from the 5a line had brain weights similar to NT littermates until postnatal day 12 (Fig. 2e), suggesting that early cortical and hippocampal development is not overtly affected by hTDP-43 expression. In contrast, a drastic decrease in brain weight was observed in symptomatic 5a mice at 24d, implying that neuronal loss rapidly occurs from P12 through the third postnatal week when the developing mouse brain expands its axonal and dendritic arborizations [22, 24] and synaptic pruning is ongoing [23]. 17d mice that survived beyond 2M also had significantly lower brain weights than NT counterparts at all ages examined; however, the observed brain atrophy was not progressive (Fig. 2f). Variability in expression level within the 17d line did not directly correlate with brain weight. We sought to determine if overt neuronal loss in iTDP-43_WT_ mice was due to apoptosis. All weaned iTDP-43_WT_ mice examined, regardless of line or phenotype, had elevated TUNEL-positive cells in cortex and hippocampus in comparison to age-matched NT mice, with the greatest number of apoptotic cells in symptomatic mice from both lines (Supplementary Fig. 3a). We determined that the cells undergoing apoptosis were neurons by double immunostaining for cleaved caspase 3 and the neuronal marker NeuN (Supplementary Fig. 3b) as well as their anatomical location in nerve cell layers of cortex and hippocampus. Overall, these results suggest that even moderate levels of wild-type hTDP-43 overexpression are extremely toxic to developing cortical and hippocampal neurons, causing severe and early neuronal loss through apoptosis.

**Fig. 3:**
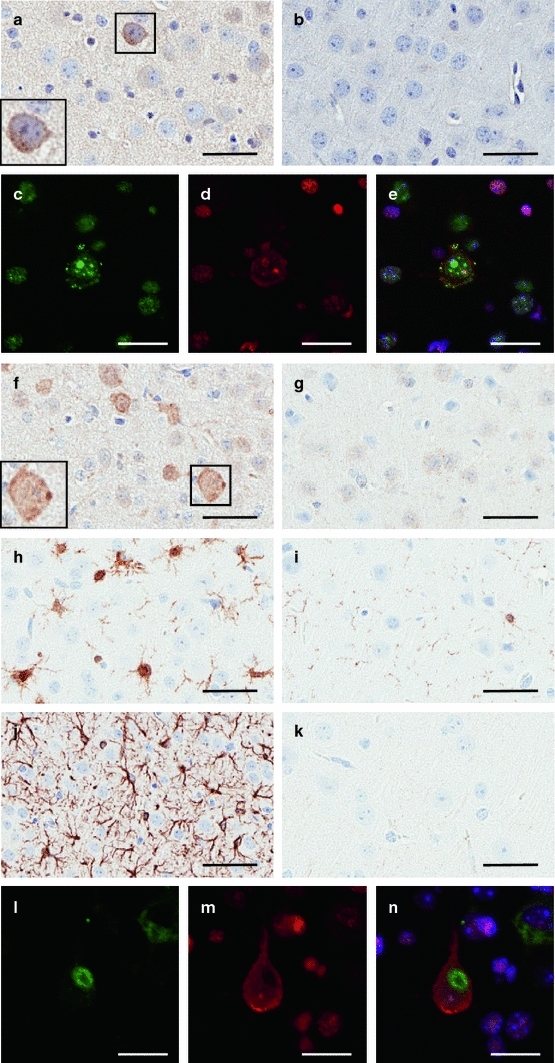
Developing neurons with hTDP-43 overexpression yield complex pathological profiles. **a**, **b** Phosphorylation of TDP-43 (pTDP-43, amino acids 403/404) was prominent in the cytoplasm of 24-day-old symptomatic 5a iT (**a**) compared to NT (**b**) mice. *Enlarged inset* (*box*) demonstrates small, cytoplasmic phospho-TDP-43 aggregates. pTDP-43 immunostaining was also observed within the nucleus, primarily surrounding nuclear bodies in neurons. **c**–**e** Fluorescent immunostaining of the cortex of 24-day-old symptomatic 5a iT for pTDP-43 (amino acids 409–410; **c**; *green*) and ubiquitin (**d**; *red*) partially co-localize (**e**; *yellow*). DAPI (**e**; *blue*) represents nuclear staining. **f**, **g** Cortical immunohistochemical staining for ubiquitin in 24-day-old symptomatic 5a iT mice (**f**) shows a substantial increase in ubiquitination when compared to NT (**g**) mice. *Enlarged inset* (*box*) demonstrates occasional cytoplasmic ubiquitin aggregates. **h**, **i** Microgliosis (Iba1) is apparent in 24-day-old symptomatic 5a iT mice (**h**) when compared to NT (**i**) mice. **j**, **k** Cortical immunohistochemical staining for astrocytosis (GFAP) in 24-day-old symptomatic 5a iT mice (**j**) shows abundant reactive astrocytes when compared to NT (**k**) mice. **l**–**n** Fluorescent immunostaining of the cortex of 24-day-old symptomatic 5a iT for the mitochondrial marker, COX-IV (**l**; *green*), and ubiquitin (**m**; *red*) demonstrates a large perinuclear COX-IV aggregate in a neuron with increased ubiquitination (**n**). DAPI (**n**; *blue*) represents nuclear staining. Similar results were obtained in line 17d summarized in Supplementary Table 2. The *bar* represents 100 μm in **a**, **b**, **f**–**k** and 20 μm in **c**–**e**, **l**–**n**

Histological examination was performed on both founder lines. We compared symptomatic 5a and 17d mice to 17d mice without an observable phenotype at 2M. Approximately 11 % of cortical neurons in symptomatic mice from the 5a line contained small (<1 μm) punctate cytoplasmic inclusions when probed with phosphorylation-specific TDP-43 antibodies that were absent in NT mice (pS403/404, Fig. 3a, b; pS409/410, Fig. 3c, e). Most inclusions from the 17d line were also cytoplasmic, but the mice that survived to 2M had pS403/404-positive-TDP-43 accumulations in the nucleus, particularly associated with nuclear bodies (Supplementary Fig. 4). In addition to TDP-43 inclusions, iTDP-43_WT_ mice of both founder lines, regardless of phenotype, had a general increase in ubiquitin immunoreactivity predominantly located in neuronal perikarya and in neuritic processes compared to NT mice (Fig. 3d, f, g and Supplementary Fig. 4). In some cases, ubiquitin accumulated in punctate inclusions (Fig. 3f, inset). Although pS409/410-TDP-43 and increased ubiquitin immunoreactivity were both frequent, the two co-localized in only about 50 % of the structures (Fig. 3c–e). iTDP-43_WT_ mice from both founder lines showed extensive reactive microgliosis (Iba-1, Fig. 3h–i and Supplementary Fig. 4) and astrocytosis (GFAP, Fig. 3j, k, Supplementary Fig. 4) compared to NT mice; symptomatic mice displayed the most robust gliosis. We also identified eosinophilic aggregates within cortical and hippocampal neuronal perikarya that were positive for the mitochondrial marker COX-IV (Supplementary Fig. 5). While the eosinophilic aggregates were similar to those previously described by our group and others in constitutive transgenic TDP-43 mice, they were smaller and less frequent than those observed in the brainstem and spinal cord of constitutive wild-type TDP-43 transgenic mice (TDP-43_PrP_) [33, 43]. Ultrastructurally, the juxtanuclear aggregates in the iTDP-43_WT_ mice were composed of abnormal mitochondria (Supplementary Fig. 5), similar to that observed in the spinal cord and brainstem of TDP-43_PrP_ mice. Co-localization experiments demonstrated neurons with mitochondrial aggregates often had increased ubiquitin immunoreactivity (Fig. 3l–n), but the mitochondrial aggregates themselves were not ubiquitinated. The mitochondrial aggregates were only present in iTDP-43_WT_ mice at 2M of age or younger. Symptomatic mice from both founder lines showed the highest frequency of mitochondrial aggregates (Supplementary Fig. 5). In summary, the pathological profiles of iTDP-43_WT_ mice with hTDP-43 overexpression during early development is complex and exhibits only limited features observed in human TDP-43 proteinopathies.

### Induction of hTDP-43 expression after weaning prevents early lethality phenotype

Given that hTDP-43 has been proposed to play crucial roles in development, we sought to determine what features of the phenotype observed in iTDP-43_WT_ mice may be due to the impact of hTDP-43 overexpression in the developing forebrain as opposed to the TDP-43 mediated proteinopathy. We utilized the conditional nature of this model to suppress hTDP-43 expression during early development with the introduction of doxycycline into the diet (Supplementary Fig. 1). We focused our suppression studies on line 5a, as this line had the most severe phenotype. hTDP-43 expression was suppressed in iTDP-43_WT_ mice from line 5a from conception until the mice reached weaning age (21d), when cortical neurons have normally matured and display extensively developed dendritic arborizations and synaptogenesis has peaked [22, 24]. At weaning, doxycycline was removed from the diet of the iTDP-43 mice to allow hTDP-43 expression; we term these mice diTDP-43_WT_.

**Fig. 4:**
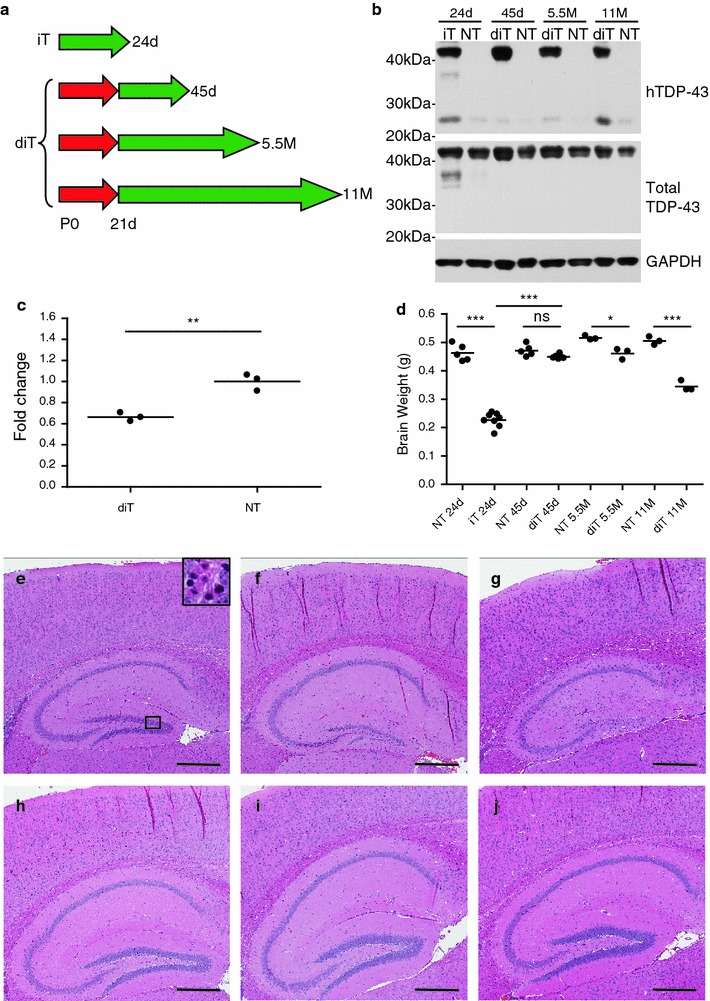
hTDP-43 induction in adult neurons overcomes early lethality and results in a progressive neurodegeneration (Line 5a). **a** 5a iTDP-43_WT_ mice (iT) which continually expressed hTDP-43 were compared to 5a diTDP-43_WT_ mice (diT) in which hTDP-43 was suppressed with doxycycline until weaning. Duration at hTDP-43 expression is shown with *green arrows*. Duration of hTDP-43 suppression is shown with *red arrows*. Final age of mice at analysis is listed at the end of each *arrow*. **b** Western blot of brain lysates from a 24-day-old iT mouse and diT mice at 45 days, 5.5 months, 11 months, and NT controls shows that hTDP-43 levels were similar between iT mice and 45d and 11 M diT mice in which hTDP-43 had been induced post-weaning. There was approximately 20 % decrease in hTDP-43 in 5.5M diT mice. Increased lower molecular weight hTDP-43 products were observed in the 24-day-old 5a iT mice at ~35 and ~25 kD, the latter was also observed in the diT 11M old mice. Total TDP-43 levels were equivalent across iT and diT mice. GAPDH was used as a loading control. **c** Quantitative real time PCR using murine-specific TDP-43 primers demonstrates reduced expression of endogenous TDP-43 in 45-day-old diT mice in the cortex relative to NT littermates (*n* = 3). **d** Whole brain weight comparison shows 45-day-old diT mice to be equivalent to NT mice and significantly greater than iT mice. However, diT brain weight decreases with age and is significantly less than NT mice by 5.5 months. **e**–**j** H&E staining of diT (**e**–**g**) and NT (**h**–**j**) mice at 45 days (**e**, **h**), 5.5 months (**f**, **i**), and 11 months (**g**, **j**) reveals progressive dentate gyrus ablation and hippocampal formation atrophy in addition to cortical thinning at 11 months. Statistical analysis was assessed by Student’s *t* test in **c**, **d**. The *bar* represents 500 μm. ***P < 0.001, **P < 0.01, *P < 0.05, *ns* no significance

None of the diTDP-43_WT_ mice (*n* = 16) showed reduced spontaneous activity, weight loss, or poor grooming, which were the phenotypes observed in 70 % of 5a iTDP-43_WT_ mice with continuous hTDP-43 expression. In addition, diTDP-43_WT_ mice had no decrease in survival, with the oldest animal without an observable phenotype at 12M. To directly compare pathology between diTDP-43_WT_ and symptomatic iTDP-43_WT_ 5a mice, we aged a cohort of diTDP-43_WT_ mice for 24d after induction of the hTDP-43 at weaning (final age 45d). We chose this duration of expression because 70 % of 5a iTDP-43_WT_ mice with continuous expression of hTDP-43 are moribund and must be euthanized by postnatal day 24; therefore, both groups of mice expressed the transgene for approximately the same length of time. We also aged additional cohorts of diTDP-43_WT_ mice following induction of hTDP-43 expression to 5.5M and 11M to determine how the impact of TDP-43 overexpression in the mature forebrain advances with age (Fig. 4a).

Biochemical examination revealed no difference in full-length hTDP-43 or total TDP-43 expression between iTDP-43_WT_ and diTDP-43_WT_ mice at 45d and 11M (Fig. 4b). The 5.5M diTDP-43_WT_ mice exhibited a 20 % decrease in hTDP-43. The symptomatic iTDP-43_WT_ mice had more low molecular weight species of hTDP-43 at around 35 and 25 kDa, with the latter also observed at 11M in the diTDP-43_WT_ mice. As in the iTDP-43_WT_ 17d mice, mTDP-43 RNA levels within the cortex of diTDP-43_WT_ (5a) mice were down-regulated in response to overexpression of hTDP-43 (0.664 ± 0.023; *P* = 0.003; Fig. 4c). Hippocampal mTDP-43 RNA levels were difficult to assess as the extensive atrophy within this region prevented a clear delineation from surrounding tissue.

### hTDP-43 induction after weaning produces key pathological features of FTLD-TDP

While neuronal loss is a salient feature of FTLD-TDP and other TDP-43 proteinopathies, the marked atrophy of the cortex and near complete ablation of the hippocampus that we observed in young iTDP-43_WT_ mice with continuous expression of hTDP-43 is far more severe and rapid than observed in any known human TDP-43 proteinopathy. To determine if the marked brain atrophy that we observed in the iTDP-43_WT_ mice was due to hTDP-43 overexpression in the environment of the developing forebrain, we compared brain weights between iTDP-43_WT_, diTDP-43_WT_, and NT mice (Fig. 4d). Brain weights of diTDP-43_WT_ at 45 days were equivalent to NT controls and significantly increased (*P* < 0.0001) over iTDP-43_WT_ mice that were matched for duration of hTDP-43 expression. The results suggest that the brain atrophy observed in young iTDP-43_WT_ mice is due to overexpression of hTDP-43 in the developing forebrain. In contrast, induction of hTDP-43 expression within more mature weanling forebrains yields a progressive loss of brain weight that worsens with aging (Fig. 4d), reaching significance at 5.5 months (*P* = 0.010) and further declining by 11 months (*P* = 0.0003). Although no overt neurodegeneration was observed in the diTDP-43_WT_ cortex at 45 days (Fig. 4e, Supplementary Fig. 6a), apoptotic bodies were detected, predominantly in the dentate gyrus (Fig. 4e, inset). The hippocampal formation in the diTDP-43_WT_ mice at 45d onward was significantly atrophied compared to NT controls (Fig. 4e–j, Supplementary Fig. 6b). Hippocampal atrophy progressed with age and became severe at 11M at which point it was accompanied by significant cortical atrophy (Supplementary Fig. 6). Striking ablation of the dentate gyrus in diTDP-43_WT_ mice became apparent by 5.5 months and progressed by 11 months (Fig. 4e–j). The dentate gyrus had a relatively high quantity of TUNEL-positive cells (Supplementary Fig. 7), correlating with this region having the most striking neuronal loss (Fig. 4e–j).

**Fig. 5:**
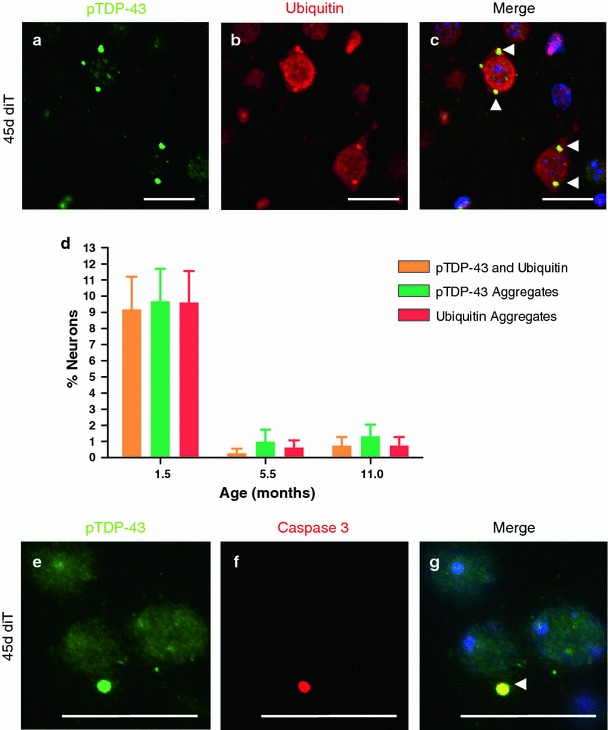
hTDP-43 expression after weaning in diTDP-43_WT_ (diT) mice from Line 5a produces salient neuropathological features of FTLD-TDP. **a**–**c** 45-day diT cortical tissue fluorescently immunostained for phospho-TDP-43 at amino acids 409–410 (**a**; *green*) and ubiquitin (**b**; *red*). Overlay (**c**) reveals frequent ubiquitin-positive, phospho-TDP-43 aggregates (*arrowheads*) at cell processes. **d** Quantitative analysis of the percent neurons containing phospho-TDP-43 inclusions, ubiquitin inclusions, and inclusions positive for both phospho-TDP-43 and ubiquitin in 45d, 5.5M, and 11M diT mice demonstrates that phospho-TDP-43 inclusions are highest in 45d diT mice (9.7 %) and drastically decreases in 5.5M (1.0 %) and 11M (1.3 %) diT mice. The phospho-TDP-43 inclusions co-localize with ubiquitin 94.3 % of the time in 45d diT mice. **e**–**g** 45-day diT cortical tissue fluorescently immunostained for pTDP-43 (**e**; *green*) and cleaved caspase 3 (**f**; *red*). Overlay (**g**) shows that cleaved caspase 3 selectively co-localizes with pTDP-43 inclusions. The *bar* represents 20 μm. SEM shown in **d**

We evaluated the brains of diTDP-43_WT_ mice for the presence of ubiquitin- and TDP-43-positive inclusions observed in human TDP-43 proteinopathies. In contrast to that observed in the iTDP-43_WT_ mice, large (>1 um) pS409/410-TDP-43 inclusions were predominant and co-localized with ubiquitin 94 % of the time in 45d diTDP-43_WT_ mice (Fig. 5a–c). Interestingly, multiple inclusions were often found within the same neuronal cell process. These were distinct, punctate inclusions akin to the TDP-43 cytoplasmic inclusions observed in FTLD-TDP. These inclusions drastically decreased in the 5.5M and 11M diTDP-43_WT_ mice (Fig. 5d). Because inclusions decreased with age while neuronal loss increased with age, we sought to determine whether neurons containing inclusions were more susceptible to neuronal loss. Co-localization studies revealed that 92 % of pS409/410-TDP-43 inclusions were co-labeled for cleaved caspase 3 (Fig. 5e–g, Supplementary Fig. 8) in 45d diT mice.

**Fig. 6:**
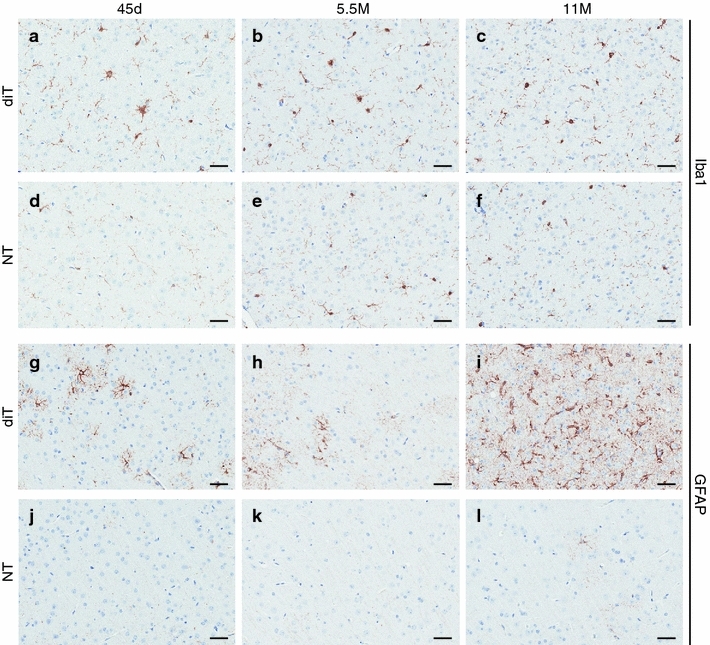
diTDP-43_WT_ mice exhibit progressive gliosis. **a**–**f** Cortical immunohistochemical staining for microgliosis (Iba1) in diT (line 5a) (**a**–**c**) and NT (**d**–**f**) mice at 45 days (**a**, **d**), 5.5 months (**b**, **e**), and 11 months (**c**, **f**) exhibits a moderate increase in reactive microglia with age in diT mice compared to NT mice. **g**–**l** Cortical immunohistochemical staining for astrocytosis (GFAP) in diT (line 5a) (**g**–**i**) and NT (**j**–**l**) mice at 45 days (**g**, **j**), 5.5 months (**h**, **k**), and 11 months (**i**, **l**) reveals a striking increase in reactive astrocytes in 11-month diT mice. The *bar* represents 100 μm

Further immunohistochemical analyses revealed that the number of cells expressing hTDP-43 within the nucleus of diTDP-43_WT_ mice decreased with age (Supplementary Fig. 9), likely reflecting the selective loss of those neurons. Diffuse cytoplasmic hTDP-43 immunoreactivity increased with age in diTDP-43_WT_ mice (Supplementary Fig. 9). diTDP-43_WT_ mice showed a moderate increase in activated microglia with age (Fig. 6a–f), while reactive astrocytes dramatically increased at 11 months (Fig. 6g–l) when compared to NT mice. In summary, the pathological profile of diTDP-43_WT_ mice with hTDP-43 overexpression in the mature forebrain shared similarities with that observed in human TDP-43 proteinopathies.

**Fig. 7:**
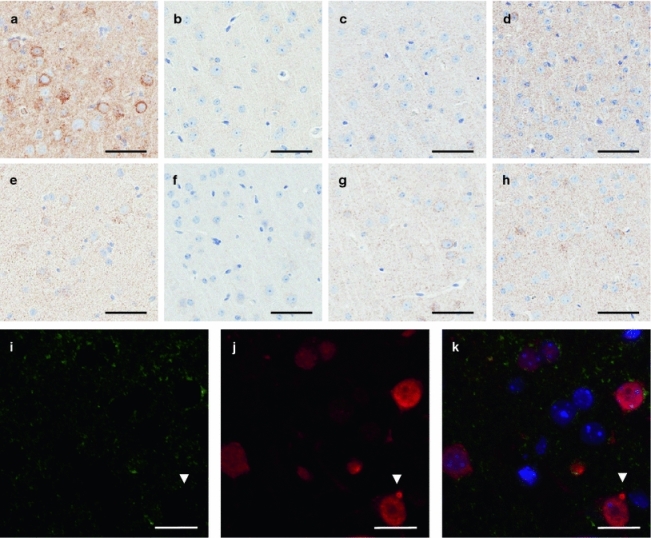
Mitochondrial aggregates are absent in diTDP-43_WT_ mice. Cortical tissue of symptomatic 5a iT (**a**), diT (**b**–**d**), and NT (**e**–**h**) mice at 24 days (**a**, **e**), 45 days (**b**, **f**), 5.5 months (**c**, **g**), and 11 months (**d**, **h**) demonstrates COX-IV immunopositive aggregates within the iT mice that are absent from diT and NT mice at all ages. **i**–**k** Fluorescent immunostaining of the cortex in 45-day-old diT mice for the mitochondrial marker, COX-IV (**i**; *green*), and ubiquitin (**j**; *red*) reveals mitochondria are not aggregated even when ubiquitin-positive aggregates are present (**k**). DAPI (**k**; *blue*) represents nuclear staining. The *bar* represents 100 μm in **a**–**h** and 20 μm in **i**–**k**

Degenerating and clustered mitochondria observed in iTDP-43_WT_ mice and other TDP-43 mouse models [33, 43] are not typical of FTLD-TDP; therefore, we sought to determine if this uncharacteristic phenotype observed in iTDP-43_WT_ mice was also present in diTDP-43_WT_ mice. We were not able to find eosinophilic aggregates by H&E in diTDP-43_WT_ mice. Moreover, abnormal immunoreactivity for COX-IV observed in iTDP-43_WT_ mice (Fig. 7a) was not observed in the diTDP-43_WT_ or NT mice at any age examined (Fig. 7b–h), even in neurons with increased ubiquitin immunoreactivity (Fig. 7i–k). These results suggest that the mitochondrial abnormality observed in iTDP-43_WT_ mice is, indeed, a consequence of hTDP-43 overexpression before P21 during early neuronal development.

Because FTLD-TDP affects patients in mid to late life, we sought to validate our observations from diTDP-43_WT_ mice with induction of hTDP-43 expression at weaning in diTDP-43 mice with induction of hTDP-43 expression at 10M. diTDP-43 mice were raised from conception until 10M of age on doxycycline to suppress hTDP-43 expression. At 10M, doxycycline was removed from the diet and the diTDP-43 mice were allowed to express hTDP-43 for 24 days. These mice are termed −10M + 24d to denote suppressed for 10 months then expressed for 24 days to distinguish them from the previously described diTDP-43 mice at 45d. hTDP-43 expression was equivalent between 45d and −10M + 24d diTDP-43_WT_ mice, demonstrating that expression of the hTDP-43 transgene were similar regardless of length of doxycycline suppression (Supplementary Fig. 10a). diTDP-43_WT_ (−10M + 24d) mice formed large pS409/410-TDP-43 inclusions that co-localized with ubiquitin in >90 % of the inclusion bearing neurons, similar to that previously observed in the diTDP-43 mice at 45d (Supplementary Fig. 10b–g). This result demonstrates that pTDP-43 inclusions can readily form in adult neurons and that the loss of inclusions observed in 5.5M and 11M diTDP-43_WT_ mice following hTDP-43 induction at weaning does not reflect a general inability of aged mice to form inclusions. Histological examination of sagittal H&E sections revealed apoptotic bodies, mainly in the dentate gyrus of the diTDP-43_WT_ (−10M + 24d) mice (Supplementary Fig. 10h). Futhermore, moderate microgliosis and astrocytosis were observed in diTDP-43_WT_ (−10M + 24d) mice (Supplementary Fig. 10i, j). These neuropathological features were elevated over that observed in NT controls (Supplementary Fig. 10k–m). In total, these preliminary results suggest that hTDP-43 induction in both young adult mice (45d) and older adult mice (−10M + 24d) demonstrate characteristic features of FTLD-TDP.

## Discussion

We have demonstrated that expression of hTDP-43 during early development yields a severe and complex phenotype (see Supplementary Table 2 for summary), including aggregation of phosphorylated TDP-43 and gliosis; however, these features are also accompanied by early lethality, extensive neuronal loss at an early age, TDP-43 inclusions that lack ubiquitin immunoreactivity and mitochondrial abnormalities that are not typical of human TDP-43 proteinopathies. A subset of the iTDP-43_WT_ mice does survive until at least 12M; however, it is not uncommon for transgenic mice to show variable penetrance and survival rates, even when on an inbred background [1, 10, 11, 14]. As with prior examples, it is difficult to pinpoint the cause of this bimodal survival; however, it does not appear to be simply a matter of transgene expression. One could conceive of unlimited pathways through which this may occur including the activity of ubiquitin-proteosomal system within each pup, the ability of some pups to adjust mTDP-43 or other unknown interacting factors quicker than others, individual pup distress during pregnancy and amount of care and suckling that any one pup receives. It should be noted that pups that escape the moribund phenotype still have significantly reduced brain weight (Fig. 2f).

hTDP-43 induction in the more mature forebrain of weaned diTDP-43_WT_ mice prevented early death, spontaneous movement deficits, severe neurodegeneration and mitochondrial abnormalities. While the loss of these features is interesting, it is rather the gain of the salient features of FTLD-TDP in diTDP-43_WT_ mice over that observed in iTDP-43_WT_ mice that is the most compelling finding of our study. diTDP-43_WT_ mice showed slowly progressing neurodegeneration, progressive gliosis and punctate cytoplasmic TDP-43 inclusions with ubiquitin immunoreactivity—each of these features closely mimics that observed in human TDP-43 proteinopathies and is not akin to that observed in iTDP-43_WT_ mice (see Supplementary Table 2 for summary). TDP-43 inclusions were primarily in cortical and hippocampal neurons, a distribution that correlates with the brain regions with highest hTDP-43 expression.

We and others have previously shown that endogenous mTDP-43 is down-regulated in response to hTDP-43 overexpression [4, 19, 33, 43]. We found that this was also the case in the cortex and hippocampus of non-symptomatic 17d iT mice and in the cortex of diT 5a mice. Importantly, heterozygous TDP-43 knockout mice express TDP-43 at levels equivalent to control mice [21, 32, 42]. Recently, a report has demonstrated that TDP-43 controls its own expression via a negative feedback loop in a human cell line [4]. Our data support these results, suggesting that neurons have a compensatory mechanism to control tightly TDP-43 levels. Moreover, this feedback mechanism appears to be present at multiple stages of development. These data implies that TDP-43 expression must remain within a precise range for cellular homeostasis.

Early lethality observed in our iTDP-43_WT_ mice and other transgenic TDP-43 models continually expressing hTDP-43 [35, 41, 43] indicates that hTDP-43 overexpression either results in an extremely rapid disease course in mice or that TDP-43 plays a critical functional role during development. The latter hypothesis was supported by one of the first published reports of TDP-43 mutations [34], where chick embryos transfected with TDP-43 mutant plasmids developed abnormal limb and tail buds and only 15 % reached the normal stage of maturation. In the current report, the severe neuronal loss and brain atrophy in iTDP-43_WT_ mice continually expressing hTDP-43 occurs in the developing forebrain and the severe brain atrophy appears not to progress in the iTDP-43_WT_ animals who live beyond 2 months of age. To determine whether TDP-43 had an impact on early brain development, we exploited the conditional nature of our model by inducing hTDP-43 expression later in development after weaning. We demonstrated that all diTDP-43_WT_ developmentally suppressed mice survived without early lethality of continually expressing iTDP-43_WT_ mice. They did not have an overt phenotype up to 12M, the oldest age examined. This result is consistent with the recent report by Igaz and colleagues [19], who described a conditional TDP-43 transgenic mouse model under the control of a CaMKIIα promoter similar to our current diTDP-43_WT_ model. Igaz and colleagues generated a high expressing line (8- to 9-fold) with a defective nuclear localization signal (hTDP-43ΔNLS) as well as a low expressing (0.4- to 1.7-fold) wild type line (hTDP-43-WT) where the transgene was suppressed until postnatal day 28 with subsequent aging to 6M without lethality. In contrast to the current report, Igaz and colleagues did not explore the differences between hTDP-43 induction in the developing brain when compared with the mature brain; therefore, it is difficult to determine if continuous expression in their model would have produced the severe phenotype and early lethality we observed in continuously expressing mice. On the other hand, a constitutive model over-expressing wild type TDP-43 at twofold higher than endogenous levels under the CaMKIIα promoter has been reported to have reduced survival though not at the early time points that we have described [36].

In the present study, iTDP-43_WT_ mice developed frequent neuronal perikaryal mitochondrial aggregates in the cortex, particularly in layer V, and occasionally in the hippocampus. Our group and others have previously generated transgenic models with continuous overexpression of TDP-43 that develops mitochondrial aggregates [33, 43]. The neuronal cytoplasmic eosinophilic aggregates observed in our iTDP-43_WT_ model were much smaller than those observed in the brainstem and spinal cord of our previously reported constitutive TDP 43_PrP_ mice [33, 43]. We did not observe eosinophilic aggregates and immunohistological (COX-IV) evidence of abnormal mitochondrial clusters in the diTDP-43_WT_ mice following post-weaning induction of hTDP-43 in mature forebrain. Interestingly, Igaz et al. also did not report the occurrence of mitochondrial clusters when they induced TDP-43 overexpression at P28 in their inducible TDP-43 mice. Given our results, we suggest that Igaz et al. did not report mitochondrial clustering because they only expressed the TDP-43 transgene in their mice from P28 onward. Our data suggest that the mechanism through which TDP-43 regulates mitochondrial biology occurs during early brain development, when the brain appears to be unusually sensitive to hTDP-43 overexpression. Our current iTDP-43_WT_ lines combined with the hTDP-43 post-weaning induction strategy will likely be useful in elucidating how TDP-43 impacts mitochondrial dynamics.

The early, severe neuronal loss and atrophy, with near total ablation of the hippocampus was perhaps the most striking phenotype that we observed in iTDP-43_WT_ mice but did not observe in diTDP-43_WT_ mice. The severe neuronal loss and atrophy in iTDP-43_WT_ mice were accompanied by comparable microgliosis and astrogliosis. In contrast, diTDP-43_WT_ mice at earlier ages had equivalent brain weights as NT mice, but they had a progressive decrease in brain weight with increasing age—a degenerative phenotype that is similar to the later onset, progressive neuronal loss and gliosis that are features of human TDP-43 proteinopathies. Igaz and colleagues previously reported that post-weaning (P28) induction of hTDP-43ΔNLS (8- to 9-fold) or hTDP-43-WT (0.4- to 1.7-fold) resulted in progressive neuronal loss, which suggests that TDP-43 dysregulation in the mature forebrain results in progressive neurodegeneration regardless of expression level [19]. Furthermore, both micro- and astrogliosis increased with age in the diTDP-43_WT_ mice. These results strongly indicate that during early development neuronal populations are highly sensitive to even moderate (2- to 3-fold) levels of hTDP-43 overexpression. The underlying mechanism through which this developmental sensitivity to TDP-43 remains to be determined, but we have identified the developmental window of selective neuronal vulnerability and, therefore, we can postulate which developmental milestones might be affected. The timing of neuronal loss during the third postnatal week suggests that neurogenesis and synaptogenesis are not markedly affected by hTDP-43 overexpression. Instead, the neuronal loss occurs when synaptic pruning is ongoing [23] and dendritic and axonal arbors are increasing in complexity [22, 24]. Consequently, activity-dependent plasticity may be implicated, and interestingly TDP-43 has been shown to act as an activity-responsive factor that represses translation in the dendrites of hippocampal neurons [39]. Thus, TDP-43 misregulation during this period could have profound effects on RNA metabolism, which could be highly toxic to neurons in the developing brain. In addition, the role of overexpression of hTDP-43 on mitochondrial pathology during early development may be associated with compromise of energy dynamics in the immature neurons, rendering them more prone to apoptosis. Certainly, the availability of the conditional in vivo model described herein will allow the field to uncover the basis of the sensitivity of developing neurons to TDP-43 misregulation.

While iTDP-43_WT_ mice develop cytoplasmic inclusions that have phosphorylated TDP-43, these inclusions only occasionally had ubiquitin immunoreactivity. In contrast, 45-day-old diTDP-43_WT_ mice developed distinct, punctate TDP-43 inclusions in neuronal perikarya and cell processes, which also had ubiquitin immunoreactivity. In this respect, TDP-43 aggregates in diTDP-43_WT_ mice are similar in appearance to TDP-43 neuronal cytoplasmic inclusions in FTLD-TDP. Interestingly, inclusions in diTDP-43_WT_ mice decrease in size and number with age, suggesting that neurons containing these inclusions may be preferentially lost. The hTDP-43ΔNLS model reported by Igaz and colleagues also had decrease in inclusions with time; however, the number of inclusions at peak was rare, ranging from <1 to <0.1 % depending on line [19]. In contrast, the phospho-TDP-43 and ubiquitin immunopositive neuronal inclusions in the constitutive CaMKII-TDP-43 Tg model reported by Tsai and colleagues were only mentioned in mice 6-month old, so we cannot determine when these inclusions became apparent or if they decreased with age [36]. Our results strongly suggest that phospho-TDP-43 aggregates are associated with neurotoxicity in that they have co-localization with activated caspase 3. Igaz and colleagues suggested that loss of murine TDP-43 within the nuclear compartment may render neurons in inducible TDP-43 mice susceptible to neurodegeneration [19]. Without a commercially available mTDP-43 antibody, we are unable to confirm these findings; however, RNA analysis suggests that a similar reduction of nuclear mTDP-43 occurs within our iTDP-43_WT_ mice. Importantly, human and mouse TDP-43 are highly homologous [38]. It would therefore seem surprising that a moderate (2- to 3-fold) level of human TDP-43 overexpression would be incapable of compensating for the nuclear loss of mTDP-43. Without the availability of an hTDP-43 knockin mouse, it is difficult to assess with certainty that the human TDP-43 can compensate for murine TDP-43 in vivo.

In generating iTDP-43_WT_ mice with forebrain expression, we sought to recapitulate features of FTLD-TDP and other TDP-43 proteinopathies, but the conditional nature of the model has also allowed us to determine the impact of TDP-43 induction at different developmental periods on the ensuing pathologic phenotype. Consequently, we report for the first time that the developing brain is more sensitive to hTDP-43 overexpression than more mature brain, despite having the same ability to autoregulate endogenous mTDP-43 levels. In addition, the early, severe neuronal loss and brain atrophy in iTDP-43_WT_ mice probably has a different pathogenesis from the progressive neurodegeneration observed in diTDP-43_WT_ mice when expression of transgene is delayed. Moreover, the present results suggest that ubiquitinated, phospho-TDP-43 aggregates may themselves be neurotoxic in mature neurons. Finally and most critically, the timing of hTDP-43 overexpression certainly affects the integrity of model phenotype as it relates to FTLD-TDP. The availability of this new TDP-43 model system provides the field with a more pathologically similar transgenic mouse model for FTLD-TDP as well as a system in which the role of TDP-43 in development versus disease can now be distinguished.

## Electronic supplementary material

Below is the link to the electronic supplementary material.

Supplementary material 1 (PPT 197,251 kb)

Supplementary material 2 (DOCX 18 kb)

Supplementary material 3 (PPT 97 kb)
