# Supplementary material for: Neuronal sensitivity to TDP-43 overexpression is dependent on timing of induction
Source: Acta Neuropathol. 2012 Apr 27;123(6):807–23. doi: 10.1007/s00401-012-0979-3 (PMC3359456; doi:10.1007/s00401-012-0979-3)
Supplement: Supplementary file 2 — Supplementary material 2 (DOCX 18 kb) [file 401_2012_979_MOESM2_ESM.docx]

**Journal**

Acta Neuropathologica

**Title**

Neuronal Sensitivity to TDP-43 Overexpression is Dependent on Timing of Induction

**Authors and Affiliations**

Ashley Cannon^1^, Baoli Yang^2^, Joshua Knight^1, 3, 4^, Ian M. Farnham^1^, Yongjie Zhang^1^, Charles A. Wuertzer^1^, Simon D’Alton^1, 3, 4^, Wen-lang Lin^1^, Monica Castanedes-Casey^1^, Linda Rousseau^1^, Brittany Scott^1^, Michael Jurasic^1^, John Howard^1, 3, 4^, Xin Yu^1^, Rachel Bailey^1, 3, 4^, Matthew R. Sarkisian^3^, Dennis W. Dickson^1^, Leonard Petrucelli^1^, Jada Lewis^1, 3, 4,*^

^1^Department of Neuroscience, Mayo Clinic, Jacksonville, Florida 32224, USA

^2^Department of Obstetrics and Gynecology, University of Iowa, Iowa City, IA 52242, USA

^3^Department of Neuroscience, University of Florida, Gainesville, FL 32611, USA

^4^Center for Translational Research in Neurodegenerative Disease, University of Florida, Gainesville, FL 32611, USA

*Correspondence:

Email: jada.lewis@ufl.edu

Phone: (352) 273-9666

Fax: (352) 294-5060

**Supplementary Fig. 1** Schematic of the iTDP-43_WT_ conditional model. The full length wild-type human TDP-43 (hTDP-43) cDNA was placed behind a minimal CMV promoter, which contained five tetracycline operator elements (TRE), effectively blocking expression in single TDP-43_WT_ transgenic mice. By crossing these mice with mice expressing the tetracycline transactivator (tTA) under the calcium calmodulin kinase II alpha promoter (CaMKIIa), we created bigenic mice, termed iTDP-43_WT_ (iT), which subsequently express the hTDP-43 protein. Administration of doxycycline blocks tTA and prevents hTDP-43 expression in diTDP-43_WT_ (diT) mice.

**Supplementary Fig. 2** Symptomatic iTDP-43_WT_ (iT) mice are significantly smaller than NT mice. **a** Picture of symptomatic 5a iT mouse (left) and NT littermate (right). **b** Symptomatic iT mice from both 5a and 17d founder lines have significantly lower body weights than NT littermates. SEM shown in **b**. *** p<0.001

**Supplementary Fig. 3** Elevated apoptosis contributes to neuronal loss in iTDP-43_WT_ (iT) mice. **a** TUNEL staining of symptomatic 5a [24-days (d) old] and 17d approximately [1 month (M) old]. iT mice exhibits numerous TUNEL-positive cells (arrowheads) in the cortex, CA1, and dentate gyrus compared to non-transgenic (NT) mice (1 month old), suggesting that iT neurons are lost through increased apoptosis. **b** Double-staining with cleaved caspase 3 (brown) and NeuN (blue) demonstrates neuronal-specific apoptosis. The bar represents 100μm.

**Supplementary Fig. 4** Increased immunoreactivity for phosphorylated TDP-43 (pTDP‑43), ubiquitination (Ubi), and gliosis in iTDP-43_WT_ (iT) mice from the 17d line. Immunohistochemical analysis of the cortices of symptomatic 17d (1M) iT mice, showed elevated pTDP-43 (pS403/404) levels, showing small cytoplasmic inclusions. In contrast, asymptomatic 17d iT mice at 2M developed intranuclear accumulations of pTDP-43 around nuclear bodies. pTDP-43 immunoreactivity was absent from non-transgenic (NT) mice. Ubiquitin immunoreactivity was increased in all iT mice compared to NT mice. Although small cytoplasmic, ubiquitinated aggregates were occasionally observed in both symptomatic and asymptomatic iT mice, increased ubiquitination predominantly occurred in a diffuse pattern within the cell body. Immunostaining for microglia (Iba1) and astrocytes (GFAP) demonstrated substantial gliosis in symptomatic 17d iT (1M) mice and asymptomatic 17d iT (2M) mice compared NT (2M) mice. Location of enlarged insets are notated by the box. The bar represents 100μm.

**Supplementary Fig. 5** Mitochondrial aggregates observed in cortex of iTDP-43_WT_ (iT) mice. **a** Cortical tissue stained for H&E revealed large, eosinophilic aggregates in symptomatic iT mice from both founder lines. COX-IV immunoreactivity exhibits densely stained aggregates in a similar region as the eosinophilic aggregates in symptomatic iT mice from both founder lines as well as young, asymptomatic 17d iT mice. The changes were not observed in non-transgenic (NT) mice. **b** Electron microscopy shows a neuron with a large cluster of mitochondria. **c** Enlargement from panel (**b**, box) demonstrates that these mitochondria often have abnormal morphology and degenerating cristae. When not noted, the bar represents 100μm.

**Supplementary Fig. 6** Forebrain structures from diTDP-43_WT_ (diT) mice are significantly smaller than those from non-transgenic (NT) mice. **a** The sum width of three cortical areas (primary motor, primary sensory, and primary visual cortices) reveal a significant decrease in cortical width at 11M in diT mice in comparison to NT mice. **b** Tracings of the hippocampal formation demonstrate a significant decrease in area as early as 45d in diT mice and progressively decreases until 11M in comparison to NT mice. SEM shown. ** p<0.01, * p<0.05, ns= no significance.

**Supplementary Fig. 7** Elevated apoptosis contributes to neuronal loss in diTDP-43_WT_ (diT) 5a mice. TUNEL staining of a diT (45d) cortex, CA1, and dentate gyrus exhibit increased DNA fragmentation over that observed in NT controls. The bar represents 100μm.

**Supplementary Fig. 8** Punctate cytoplasmic inclusions are immunopositive for pTDP-43 and activated Caspase 3. **a** Quantitative analysis of the percent neurons containing pS409/410-TDP-43 aggregates, cleaved caspase 3 (C3) aggregates, and aggregates positive for both pS409/410-TDP-43 and caspase 3 in 45d, 5.5M, and 11M diT mice demonstrate that pS409/410-TDP-43 aggregates colocalize with caspase 3 92% of the time in 45d diT mice and declines with age as the pTDP-43 aggregates decline. **b** Quantitative analysis of the percent neurons containing diffuse caspase 3 (C3) immunoreactivity shows a progressive increase of diffuse caspase 3 as diTDP-43 mice age; however, this does not correlate with the appearance of pTDP-43 aggregates. SEM shown.

**Supplementary Fig. 9** hTDP-43 is distributed in both the nucleus and the cytoplasm in diTDP-43_WT_ (diT) mice. Cortical tissue from 45d, 5.5M, and 11M diT and NT mice immunostained for hTDP-43 exhibit some cytoplasmic redistribution in aged diT mice. Number of neurons immunostaining for hTDP-43 decreases with age, likely reflecting loss of that neuronal population. The bar represents 100μm.

**Supplementary Fig. 10** diTDP-43_WT_ (diT) mice demonstrate similar features when hTDP-43 is induced at weaning compared in older adult mice. **a** Western blot of brain lysates from 45d diT, -10M-24d diT, 11 month diT, and NT controls show that hTDP-43 levels were similar between 45d and -10M+24d diT mice, which have a similar length of hTDP-43 expression (24d). **b-g** 45d and -10M+24d diT cortical tissue fluorescently immunostained for pTDP-43 at amino acids 409-410 (**b,e**; green) and ubiquitin (**c,f**; red). Overlay (**d,g**) reveals analogous ubiquitin-positive, pTDP-43 aggregates in both groups of diT mice. **h-m** Histological comparison of -10M+24d diT (**h-j**) and NT (**k-m**) mice. **h**,**k** Sagittal H&E sections show that the diT mice have apoptotic bodies (**h**, inset) in the dentate gyrus. Cortical immunohistochemical staining for microgliosis (Iba1; **i,l**) and astrocytosis (GFAP; **j,m**) reveal a moderate increase in reactive gliosis in the -10M+24d diT mice. The bar represents 10 μm in (**b-g**), 250 μm in (**h,k)**, and 100μm in (**i-j, l-m**).
