# Supplementary material for: Neuronal sensitivity to TDP-43 overexpression is dependent on timing of induction
Source: Acta Neuropathol. 2012 Apr 27;123(6):807–23. doi: 10.1007/s00401-012-0979-3 (PMC3359456; doi:10.1007/s00401-012-0979-3)
Supplement: Supplementary file 3 — Supplementary material 3 (PPT 97 kb) [file 401_2012_979_MOESM3_ESM.ppt]

## Slide 1
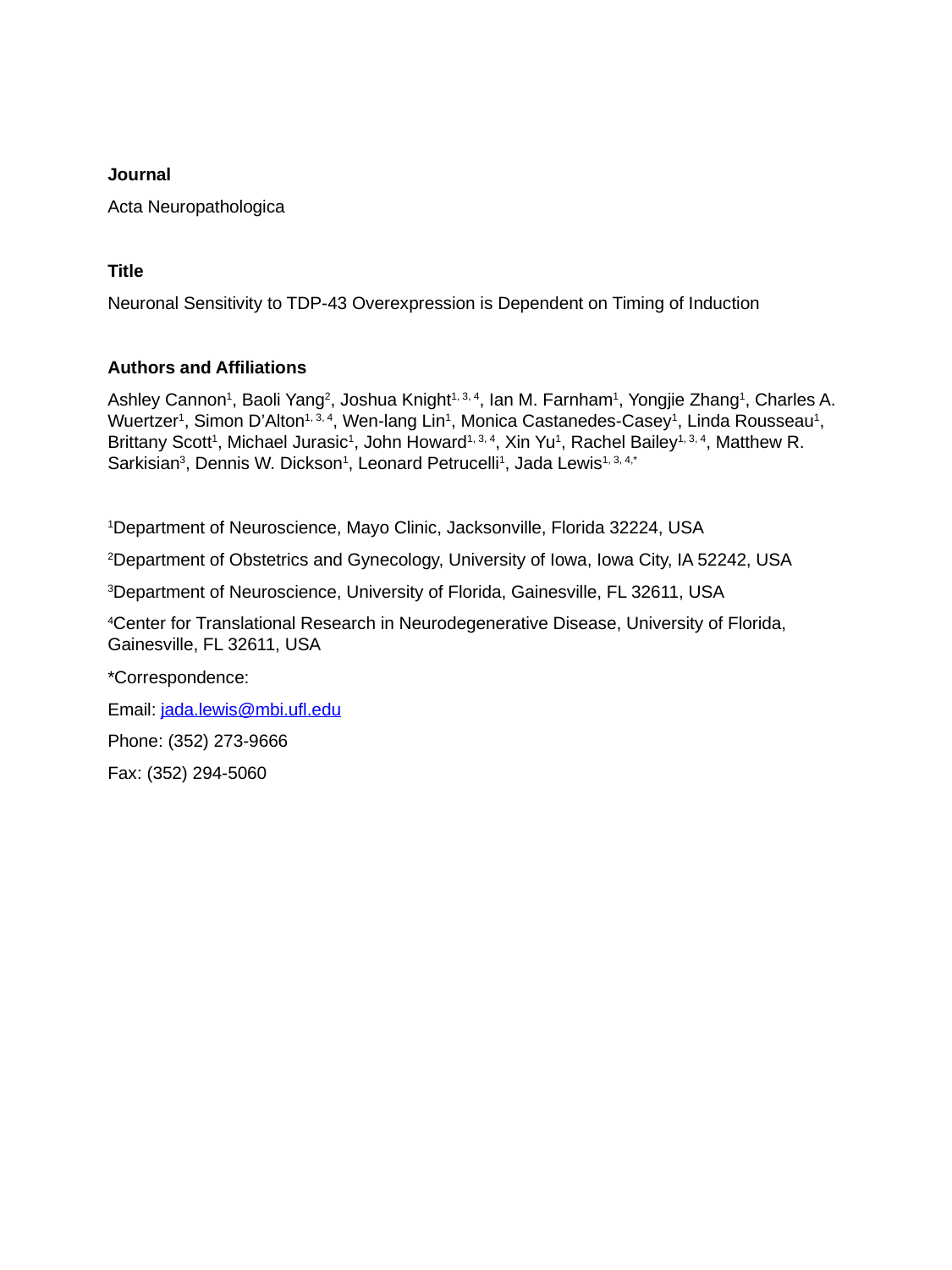

Journal
Acta Neuropathologica
Title
Neuronal Sensitivity to TDP-43 Overexpression is Dependent on Timing of Induction
Authors and Affiliations
Ashley Cannon1, Baoli Yang2, Joshua Knight1, 3, 4, Ian M. Farnham1, Yongjie Zhang1, Charles A. Wuertzer1, Simon D’Alton1, 3, 4, Wen-lang Lin1, Monica Castanedes-Casey1, Linda Rousseau1, Brittany Scott1, Michael Jurasic1, John Howard1, 3, 4, Xin Yu1, Rachel Bailey1, 3, 4, Matthew R. Sarkisian3, Dennis W. Dickson1, Leonard Petrucelli1, Jada Lewis1, 3, 4,*
1Department of Neuroscience, Mayo Clinic, Jacksonville, Florida 32224, USA
2Department of Obstetrics and Gynecology, University of Iowa, Iowa City, IA 52242, USA
3Department of Neuroscience, University of Florida, Gainesville, FL 32611, USA
4Center for Translational Research in Neurodegenerative Disease, University of Florida, Gainesville, FL 32611, USA
*Correspondence:
Email: jada.lewis@mbi.ufl.edu
Phone: (352) 273-9666
Fax: (352) 294-5060

## Slide 2
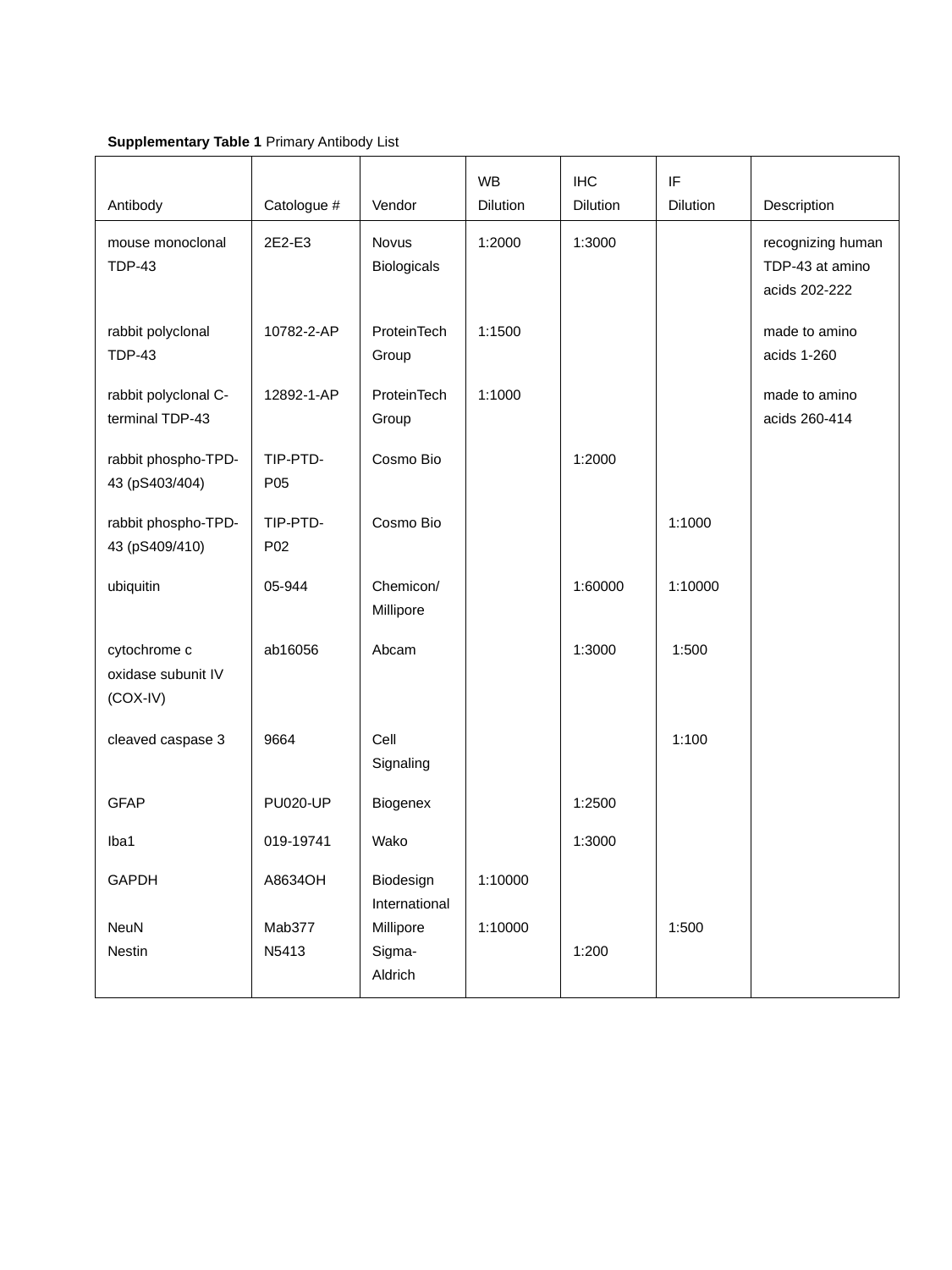

| Supplementary Table 1 Primary Antibody List | | | | | | |
| --- | --- | --- | --- | --- | --- | --- |
| Antibody | Catologue # | Vendor | WB Dilution | IHC Dilution | IF Dilution | Description |
| mouse monoclonal TDP-43 | 2E2-E3 | Novus Biologicals | 1:2000 | 1:3000 | | recognizing human TDP-43 at amino acids 202-222 |
| rabbit polyclonal TDP-43 | 10782-2-AP | ProteinTech Group | 1:1500 | | | made to amino acids 1-260 |
| rabbit polyclonal C-terminal TDP-43 | 12892-1-AP | ProteinTech Group | 1:1000 | | | made to amino acids 260-414 |
| rabbit phospho-TPD-43 (pS403/404) | TIP-PTD-P05 | Cosmo Bio | | 1:2000 | | |
| rabbit phospho-TPD-43 (pS409/410) | TIP-PTD-P02 | Cosmo Bio | | | 1:1000 | |
| ubiquitin | 05-944 | Chemicon/ Millipore | | 1:60000 | 1:10000 | |
| cytochrome c oxidase subunit IV (COX-IV) | ab16056 | Abcam | | 1:3000 | 1:500 | |
| cleaved caspase 3 | 9664 | Cell Signaling | | | 1:100 | |
| GFAP | PU020-UP | Biogenex | | 1:2500 | | |
| Iba1 | 019-19741 | Wako | | 1:3000 | | |
| GAPDH NeuN Nestin | A8634OH Mab377 N5413 | Biodesign International Millipore Sigma-Aldrich | 1:10000 1:10000 | 1:200 | 1:500 | |

## Slide 3
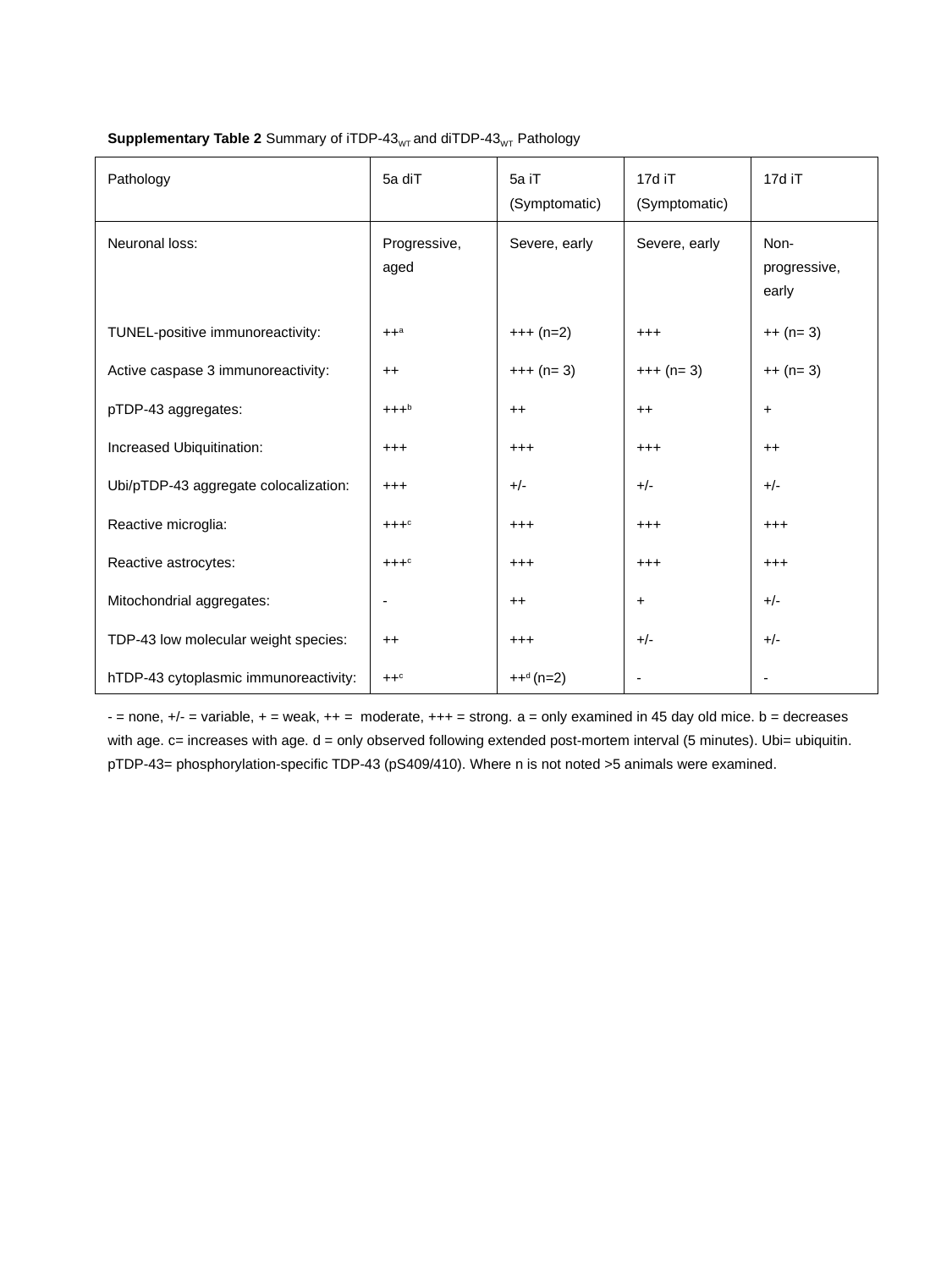

| Supplementary Table 2 Summary of iTDP-43WT and diTDP-43WT Pathology | | | | |
| --- | --- | --- | --- | --- |
| Pathology | 5a diT | 5a iT (Symptomatic) | 17d iT (Symptomatic) | 17d iT |
| Neuronal loss: | Progressive, aged | Severe, early | Severe, early | Non- progressive, early |
| TUNEL-positive immunoreactivity: | ++a | +++ (n=2) | +++ | ++ (n= 3) |
| Active caspase 3 immunoreactivity: | ++ | +++ (n= 3) | +++ (n= 3) | ++ (n= 3) |
| pTDP-43 aggregates: | +++b | ++ | ++ | + |
| Increased Ubiquitination: | +++ | +++ | +++ | ++ |
| Ubi/pTDP-43 aggregate colocalization: | +++ | +/- | +/- | +/- |
| Reactive microglia: | +++c | +++ | +++ | +++ |
| Reactive astrocytes: | +++c | +++ | +++ | +++ |
| Mitochondrial aggregates: | - | ++ | + | +/- |
| TDP-43 low molecular weight species: | ++ | +++ | +/- | +/- |
| hTDP-43 cytoplasmic immunoreactivity: | ++c | ++d (n=2) | - | - |
| - = none, +/- = variable, + = weak, ++ = moderate, +++ = strong. a = only examined in 45 day old mice. b = decreases with age. c= increases with age. d = only observed following extended post-mortem interval (5 minutes). Ubi= ubiquitin. pTDP-43= phosphorylation-specific TDP-43 (pS409/410). Where n is not noted >5 animals were examined. | | | | |
